# Supplementary material for: Historic transposon mobilisation waves create distinct pools of adaptive variants in a major crop pathogen
Source: Nat Commun. 2025 Nov 12;16:9961. doi: 10.1038/s41467-025-64944-4 (PMC12612061; doi:10.1038/s41467-025-64944-4)
Supplement: Supplementary file 1 — Supplementary Information [file 41467_2025_64944_MOESM1_ESM.pdf]

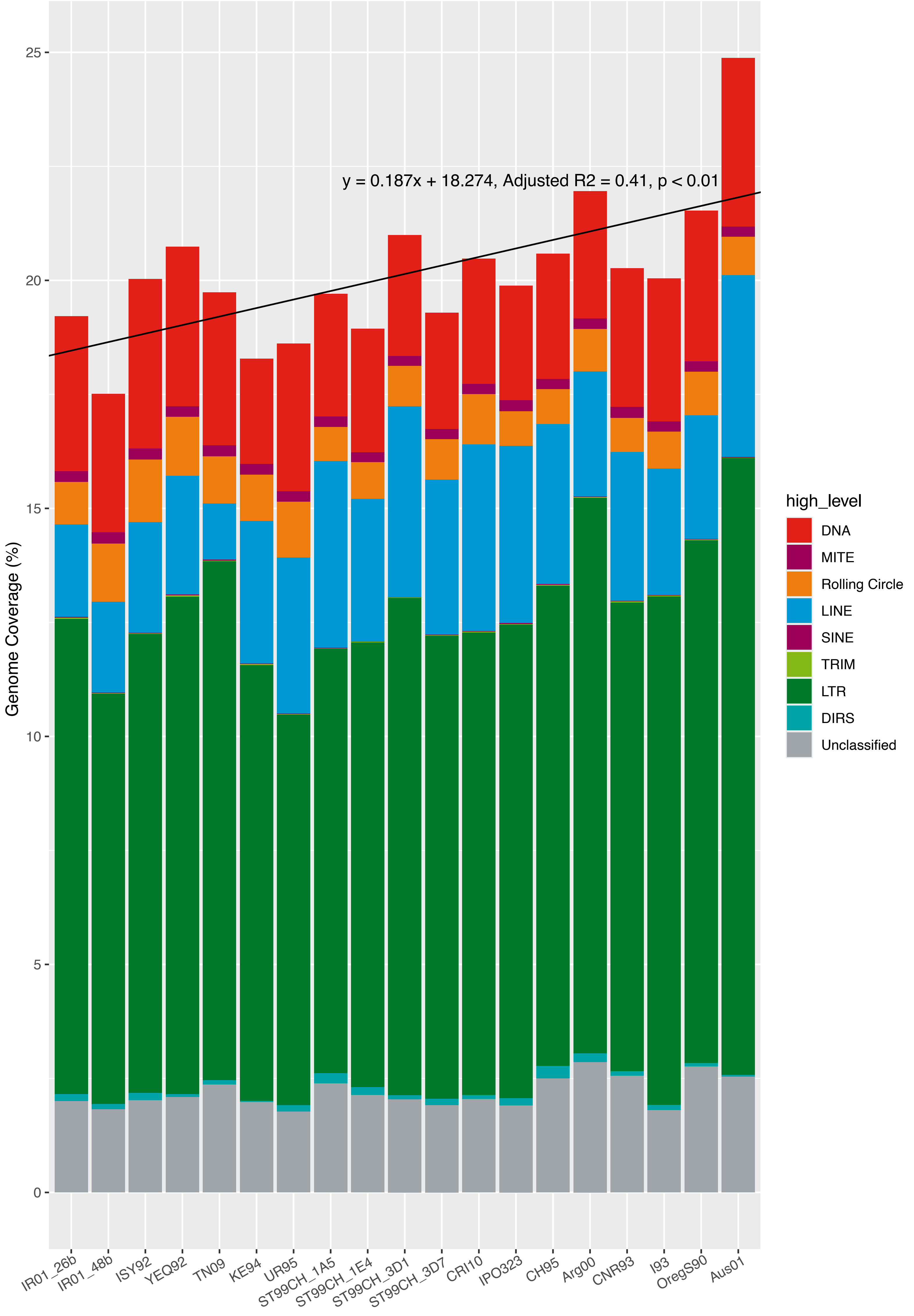

Figure S1. Transposable element content among long-read reference-quality assemblies of the Zymoseptoria tritici global panel. Isolates are ordered based on rough colonisation history, with most ancient on the left and most recent on the right of the X axis. TE content is expressed as percentage of total genome size. Black line shows linear model of total genomic TE percentage by assembly (in order of colonisation), with the equation $y=0.187x + 18.274$, $R2 = 0.41$, $p < 0.01$.

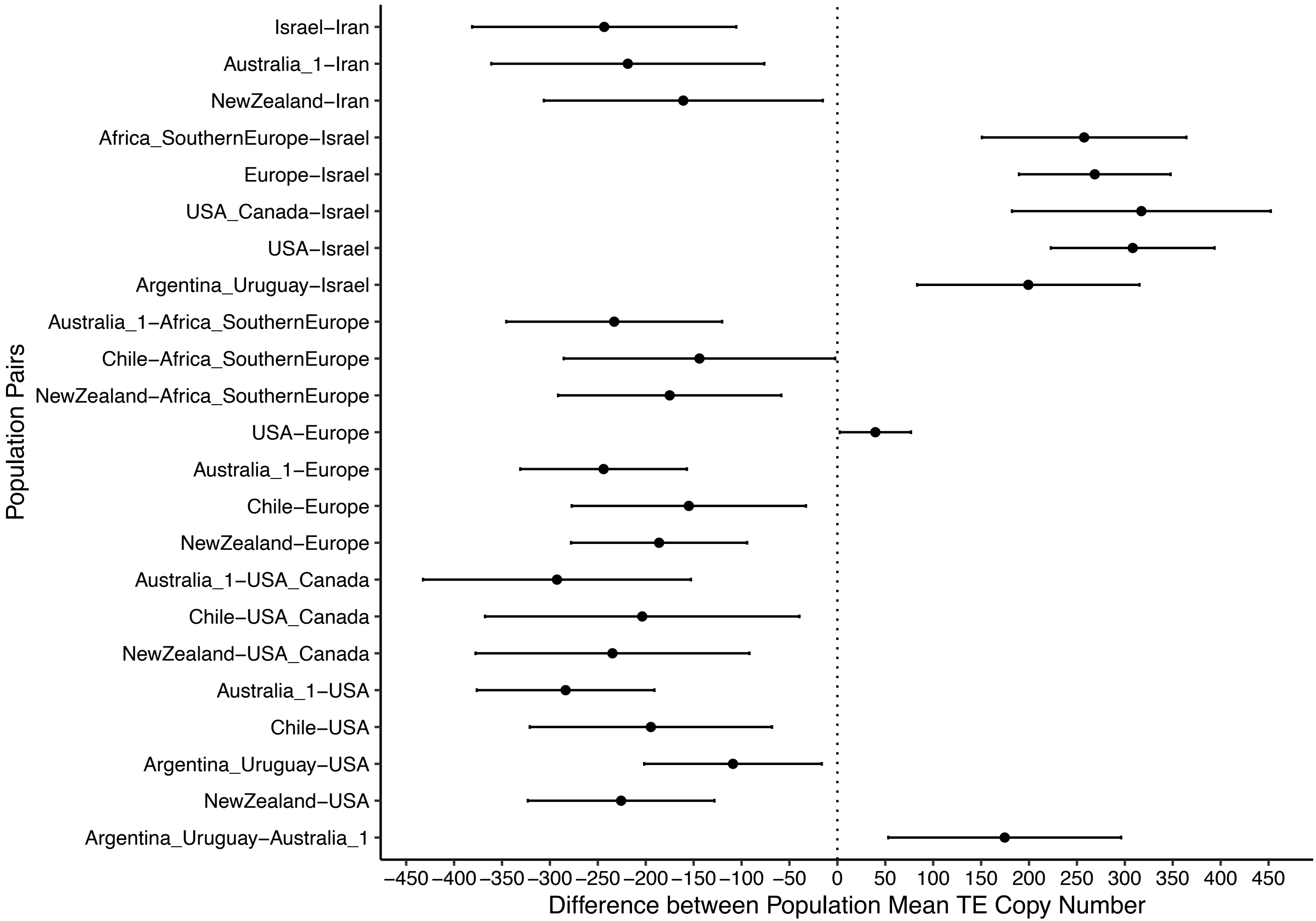

Figure S2. Significant pairwise comparisons between populations with varying TE copy numbers, calculated using Tukey Honest Significant Differences, with an adjusted P value significance cutoff $P < 0.05$. Pairwise comparisons are shown on the Y axis, and difference between population means on the X axis. Points indicate mean pairwise difference between populations, and bars indicate 95% confidence intervals. Negative changes indicate that the second population of each pair has a higher TE content, whilst positive changes indicate that the first population of each pair has a higher TE content. Population samples sizes: Iran n=16; Israel n=34; Africa & Southern Europe n=39; Europe n=1,190; Chile n=14; Argentina & Uruguay n=28; USA & Canada n=17; USA n=171; Australia_1 n=28; Australia_2 n=7; New Zealand n=25.

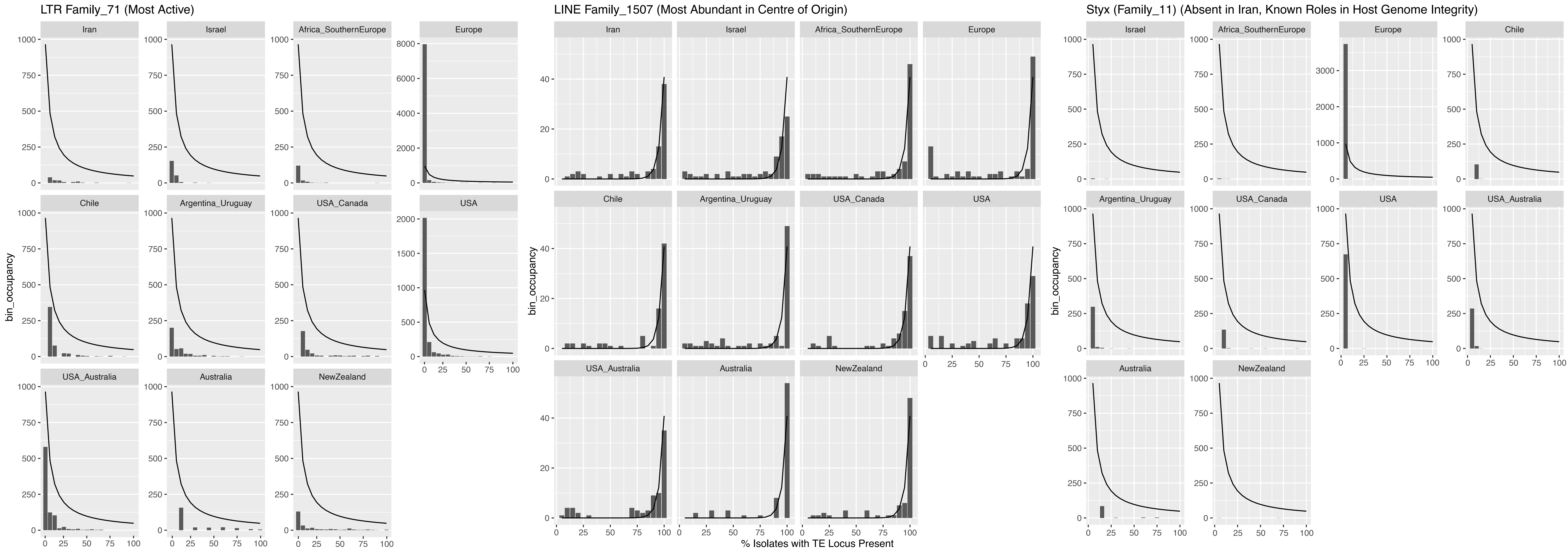

Figure S3. TE locus occupancy for the putatively most active TE family (LTR family_71), the most abundant TE family in the centre of origin (LINE family_1507), and a TE family with experimental evidence for ongoing activity (Styx family_11). X axis shows TE frequency for each locus. Y axis indicates the number of TE loci at a given frequency interval. Black lines show modelled frequency spectra based on global TE locus occupancy. Population samples sizes: Iran n=16; Israel n=34; Africa & Southern Europe n=39; Europe n=1,190; Chile n=14; Argentina & Uruguay n=28; USA & Canada n=17; USA n=171; Australia_1 n=28; Australia_2 n=7; New Zealand n=25.

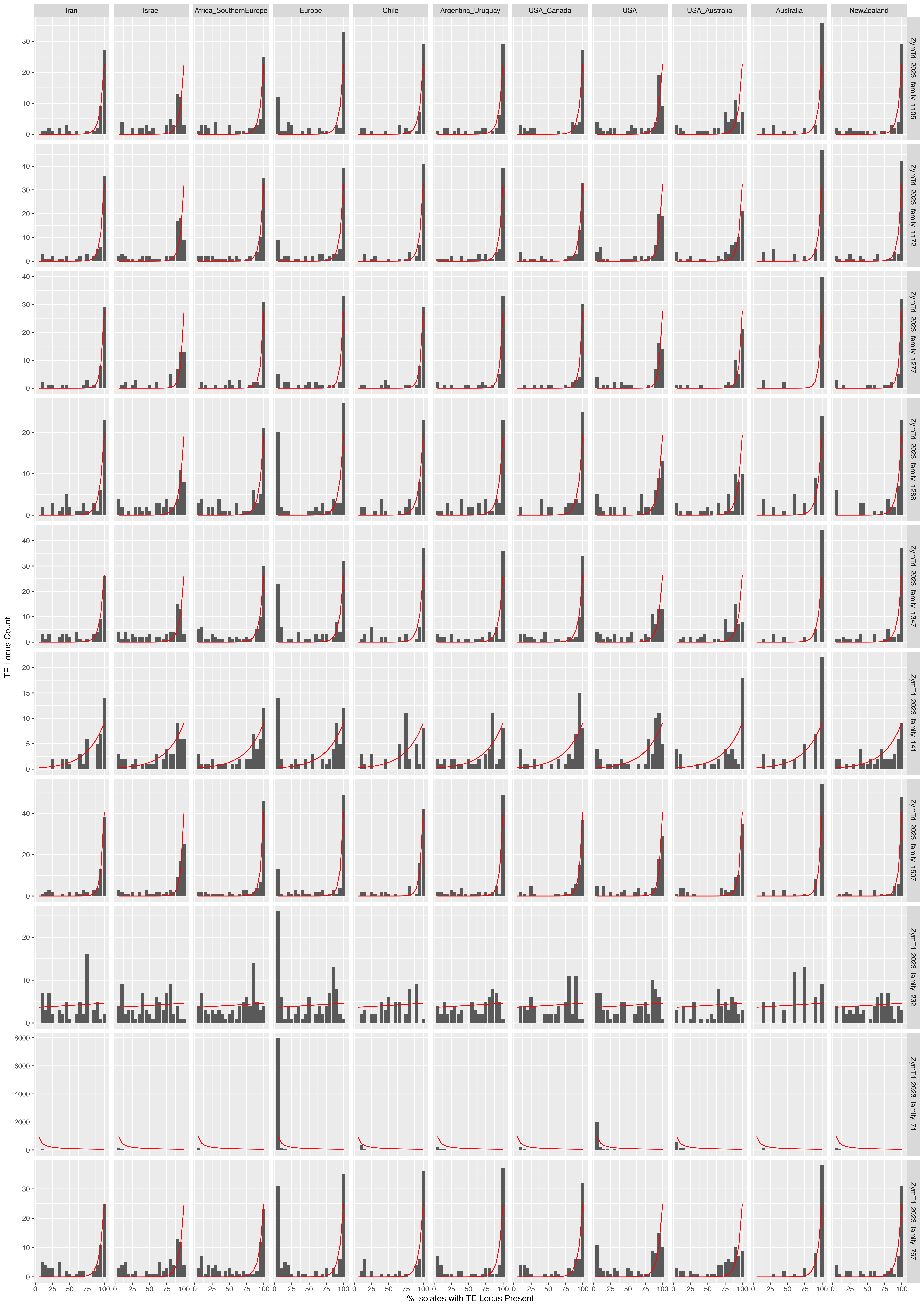

Figure S4˜ TE locus occupancy for the ten most abundant TE families in the global panel of *Z. tritici* X axis shows the percentage of isolates in a population containing a given TE locus˜ Y axis shows the locus count at a given population frequency˜ Each column shows TE frequency spectra for a given population° ordered from centre of origin to most recently derived population˜ Each row shows TE frequencies for a given TE family˜ Red lines show expected TE frequency spectra based on global panel locus occupancy˜ Population samples sizes: Iran n=16; Israel n=34; Africa & Southern Europe n=39; Europe n=1,190; Chile n=14; Argentina & Uruguay n=28; USA & Canada n=17; USA n=171; Australia_1 n=28; Australia_2 n=7; New Zealand n=25.

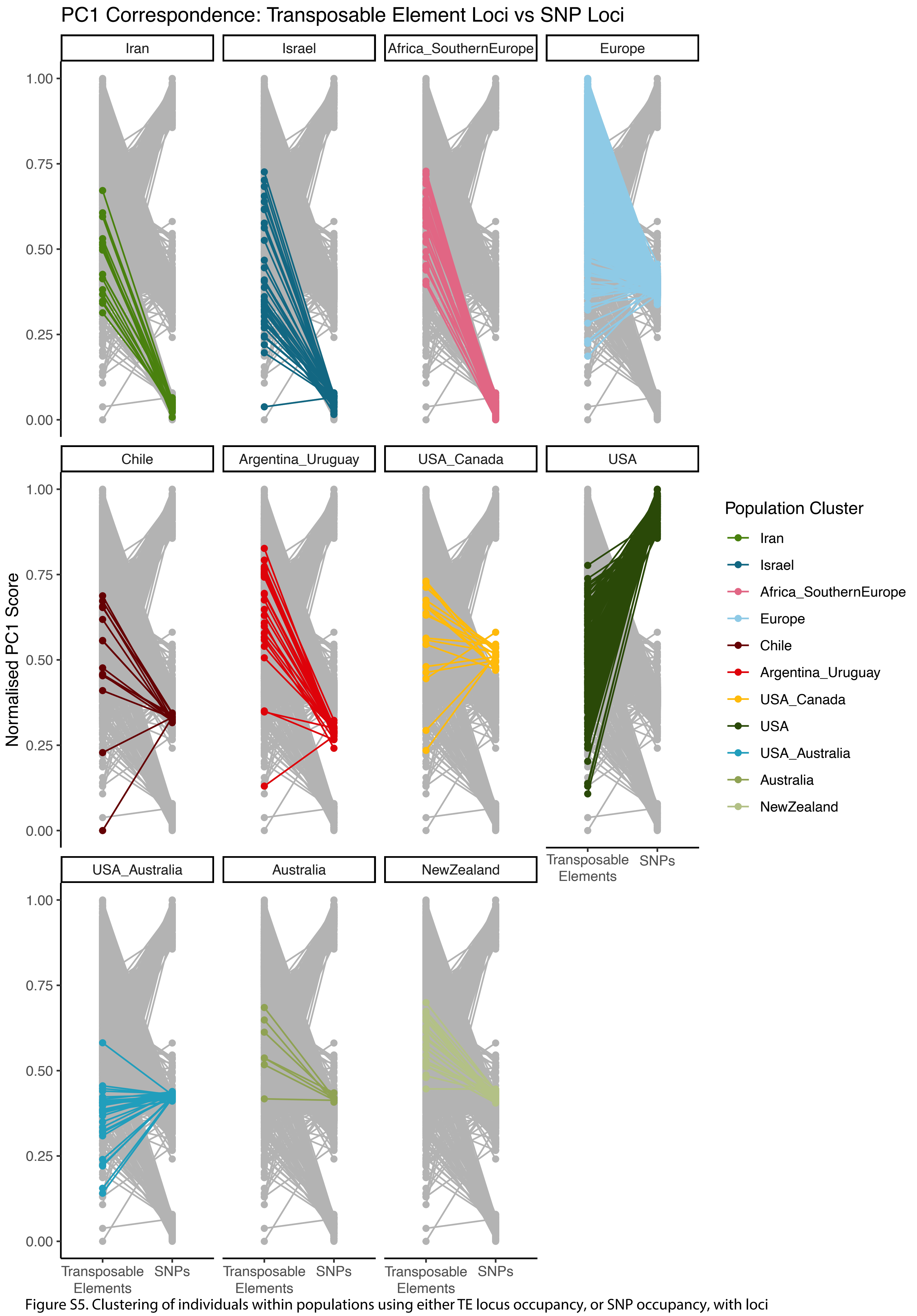

Figure S5. Clustering of individuals within populations using either TE locus occupancy, or SNP occupancy, with loci filtered to minor allele frequency > 0.05. X axis indicates whether the principal component was computed using TE loci or SNP loci. Y axis shows normalised score for the first principal component. Each dot indicates an individual in a population, with connecting lines joining the same individual when using either TEs or SNPs. Focal population is shown in colour, as indicated in the key. In each panel, the non-focal populations are shown in grey. Population samples sizes: Iran n=16; Israel n=34; Africa & Southern Europe n=39; Europe n=1,190; Chile n=14; Argentina & Uruguay n=28; USA & Canada n=17; USA n=171; Australia_1 n=28; Australia_2 n=7; New Zealand n=25.

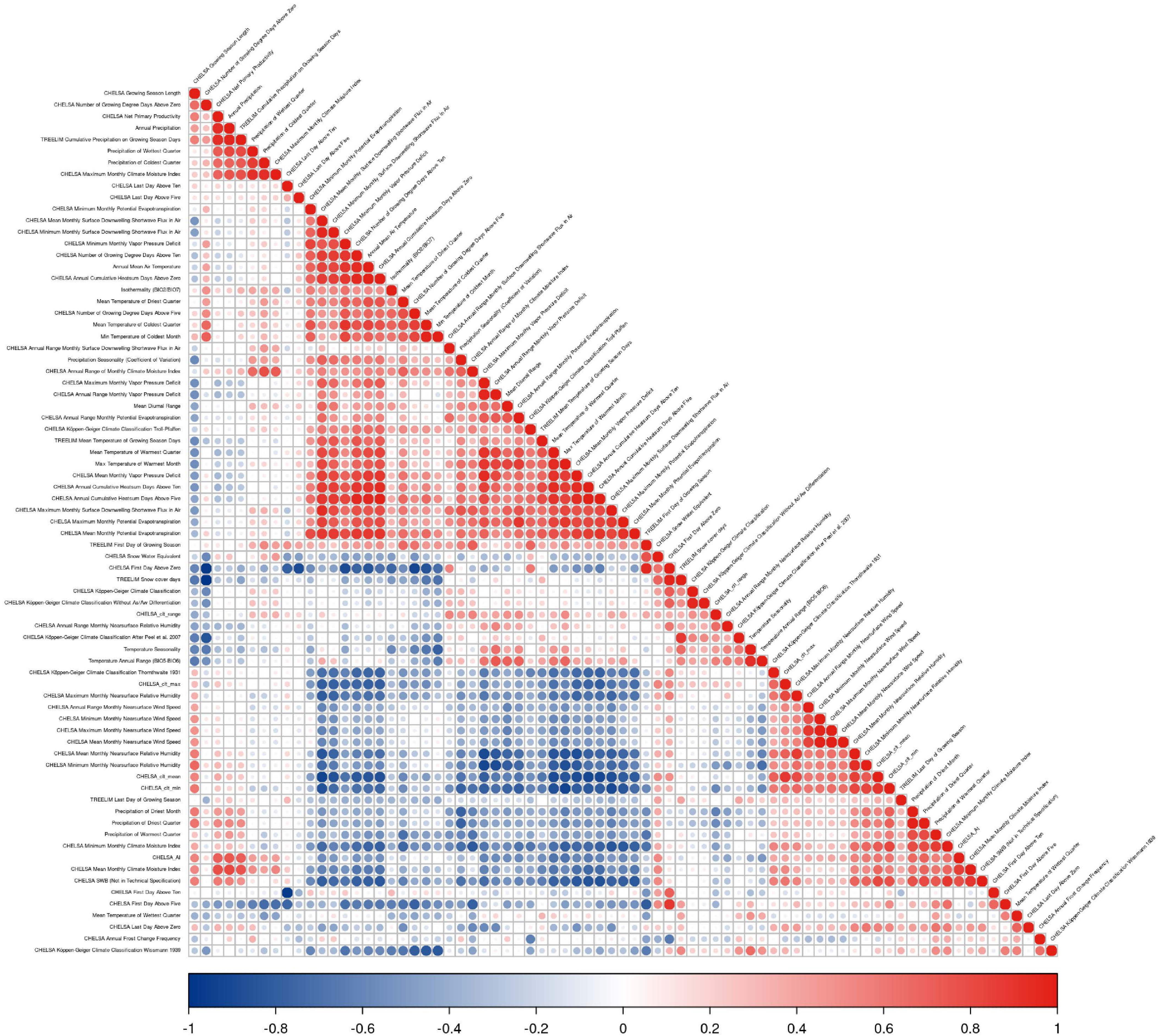

Figure S6. Correlations among bioclimatic variables. The correlation strength and direction is indicated in the key, with dark blue indicating a strong negative correlation and red indicating a strong positive correlation.
