# Supplementary material for: Historic transposon mobilisation waves create distinct pools of adaptive variants in a major crop pathogen
Source: Nat Commun. 2025 Nov 12;16:9961. doi: 10.1038/s41467-025-64944-4 (PMC12612061; doi:10.1038/s41467-025-64944-4)
Supplement: Supplementary file 2 — Description of Additional Supplementary Files [file 41467_2025_64944_MOESM2_ESM.pdf]

Description of Additional Supplementary Information

File Name: Supplementary Data 1
Description: Additional discussion on benchmarking of TE polymorphism detection tools provided within the McClintock2 framework.

File Name: Supplementary Data 2
Description: TAR archive containing a FASTA file for the pangenome assembly of Zymoseptoria tritici, along with a dictionary to map simplified FASTA headers, and a BED file containing polymorphic TE annotations for all Z. tritici isolates included in this study (including isolates that could not be assigned to a single cluster). This file has been uploaded to the Zenodo database under DOI 10.5281/zenodo.17189961 (https://doi.org/10.5281/zenodo.17189961).

File Name: Supplementary Data 3
Description: Compressed VCF containing TE presence absence polymorphism data used for GEMMA analysis to identify polymorphic TEs associated with bioclimate principal components. This file has been uploaded to the Zenodo database under DOI 10.5281/zenodo.17189961 (https://doi.org/10.5281/zenodo.17189961).

File Name: Supplementary Data 4
Description: Transposable Element Quantifications for Reference-Quality Assemblies Used for Initial TE Manual Curation

File Name: Supplementary Data 5
Description: Tukey Post-Hoc Comparisons for TE Abundance Differences Among Populations, calculated using a one-way analysis of variance. Tukey post-hoc p-values are adjusted to account for multiple comparisons.

File Name: Supplementary Data 6
Description: Transposable Element Abundance per Isolate, separated by TE classification

File Name: Supplementary Data 7
Description: Transposable Element Locus Frequency by population cluster

File Name: Supplementary Data 8
Description: Transposable Element Population Locus Quantifications

File Name: Supplementary Data 9
Description: Gene Ontology Annotations for genes proximal to candidate TEs

File Name: Supplementary Data 10
Description: 45 candidate TE loci showing evidence for local adaptation, including the populations in which they have risen to common frequency, and their position in relation to host genes. Promoter regions are defined as the region 1 kb upstream of the TSS.

File Name: Supplementary Data 11
Description: Principle Component Loadings for Clustering of Bioclimate Variables

File Name: Supplementary Data 12
Description: Isolate metadata, including the isolate name, assigned genetuc cluster, geographical location of the sampling site and inferred coordinates, sampling year, the bioproject corresponding to the sequencing data, and whether it was incorporated in the analysis

File Name: Supplementary Data 13
Description: Coordinates of predicted effectors for IPO323 from Thynne et al. (2024) in BED format

File Name: Supplementary Data 14
Description: Tables with Results of McClintock2 benchmarking analyses.
