# Supplementary material for: Historic transposon mobilisation waves create distinct pools of adaptive variants in a major crop pathogen
Source: Nat Commun. 2025 Nov 12;16:9961. doi: 10.1038/s41467-025-64944-4 (PMC12612061; doi:10.1038/s41467-025-64944-4)
Supplement: Supplementary file 3 — Supplementary Data 1 [file 41467_2025_64944_MOESM3_ESM.docx]

**Supplementary Data 1 - Benchmarking TE Detection Methods**

To facilitate our investigation, a balance must be struck between maximising TE detection and retaining a high level of confidence in resultant TEs annotations. A conservative approach is preferred, where false positive annotations are minimised at the cost of introducing some false negative annotations, such that a higher level of confidence in the resultant annotations is retained. Importantly, in the case of TEMP and TEMP2 implemented in McClintock2, reference TEs are called when there is no evidence to support TE insertion absence (appearing as a “*non-abs*” call in the annotation). In a pangenomic context, this is problematic as all annotated TEs within the pangenome will be called as present due to a lack of evidence to support TE absence, resulting in a high false positive count. In the context of our study, this behaviour is undesirable, thus we only consider the performance of TEMP and TEMP2 in detecting non-reference TE insertions.

### **Analysis 1: Mapping simulated reads with added non-reference TE insertions to an unmodified reference assembly**

For a methodology to perform perfectly, 675 TEs will be annotated in analysis 1 (639 reference TEs and 36 non-reference TEs). The proportion of missed reference TE insertions ranged from 100% (639 TEs: ngs_te_mapper) to 28.32% (180 TEs: TEFLoN). Considering methodologies that annotated at least 1 TE locus, the proportion of total reference TE insertions correctly annotated (i.e. as a reference TE of the correct TE family) ranged from 9.39% (60 TEs: ngs_te_mapper2) to 71.67% (458 TEs: TEFLoN), whilst non-reference detection varied from 8.33% (3 TEs: ngs_te_mapper, TEMP) to 72.22% (26 TEs: PoPoolationTE2). The only tool to detect false positives was PoPoolationTE2, which incorrectly annotated 25 loci as non-reference TE insertions. Incorrect TE annotations remained low among remaining methodologies, with misclassified non-reference annotations varying from 2.78% (1 non-reference TE: TEFLoN, PoPoolationTE, PoPoolationTE2) to 27.78% (10 non-reference TE: TElocate). Misclassified reference annotations varied from 0.16% (1 TE: TEFLoN, TElocate) to 0.31% (2 TEs: TEMP, TEMP2). Full annotation results are provided in supplementary data 14, and visualisation is provided in figure 1.


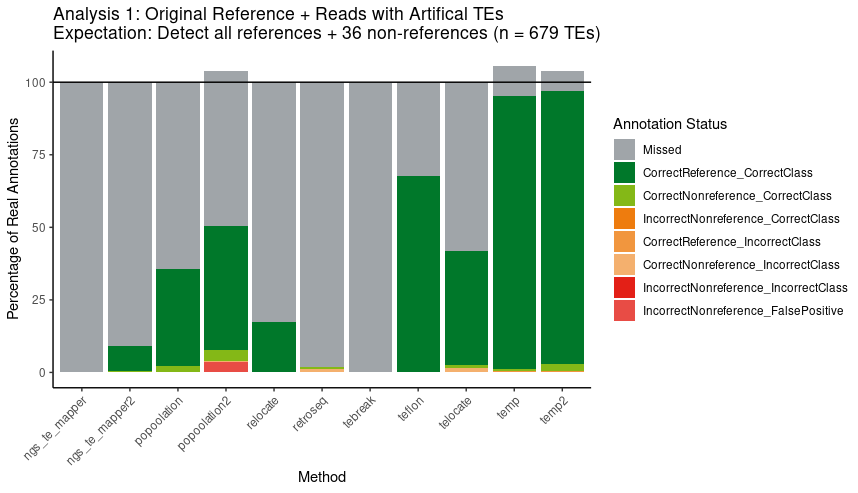


Figure 1. TE benchmarking results for analysis 1. Methodologies were able to detect >100% of the read annotations if loci were annotated multiple times, or if false positives were identified. The status of each annotated locus is indicated by the key.

### **Analysis 2: Mapping unmodified simulated reads to a reference assembly with added non-reference TE insertions**

For a methodology to perform perfectly, 639 reference TEs will be annotated in analysis 2. The proportion of missed reference TE insertions ranged from 100% (639 TEs: ngs_te_mapper) to 41.47% (265 TEs: TEFLoN). The proportion of reference TEs that were correctly annotated (i.e. as a reference TE of the correct TE family) ranged from 10.02% (64 TEs: ngs_te_mapper2) to 58.53% (374 TEs: TEFLoN). False positive non-reference TEs were annotated by PoPoolationTE2 (28 TEs) and RetroSeq (1 TE). Both TEMP2 and RetroSeq also incorrectly annotated 1 reference TE locus as non-reference, but assigned the correct classification. PoPoolationTE and TElocate each misclassified 1 reference TE locus. Full annotation results are provided in supplementary data 14, and visualisation is provided in figure 2.


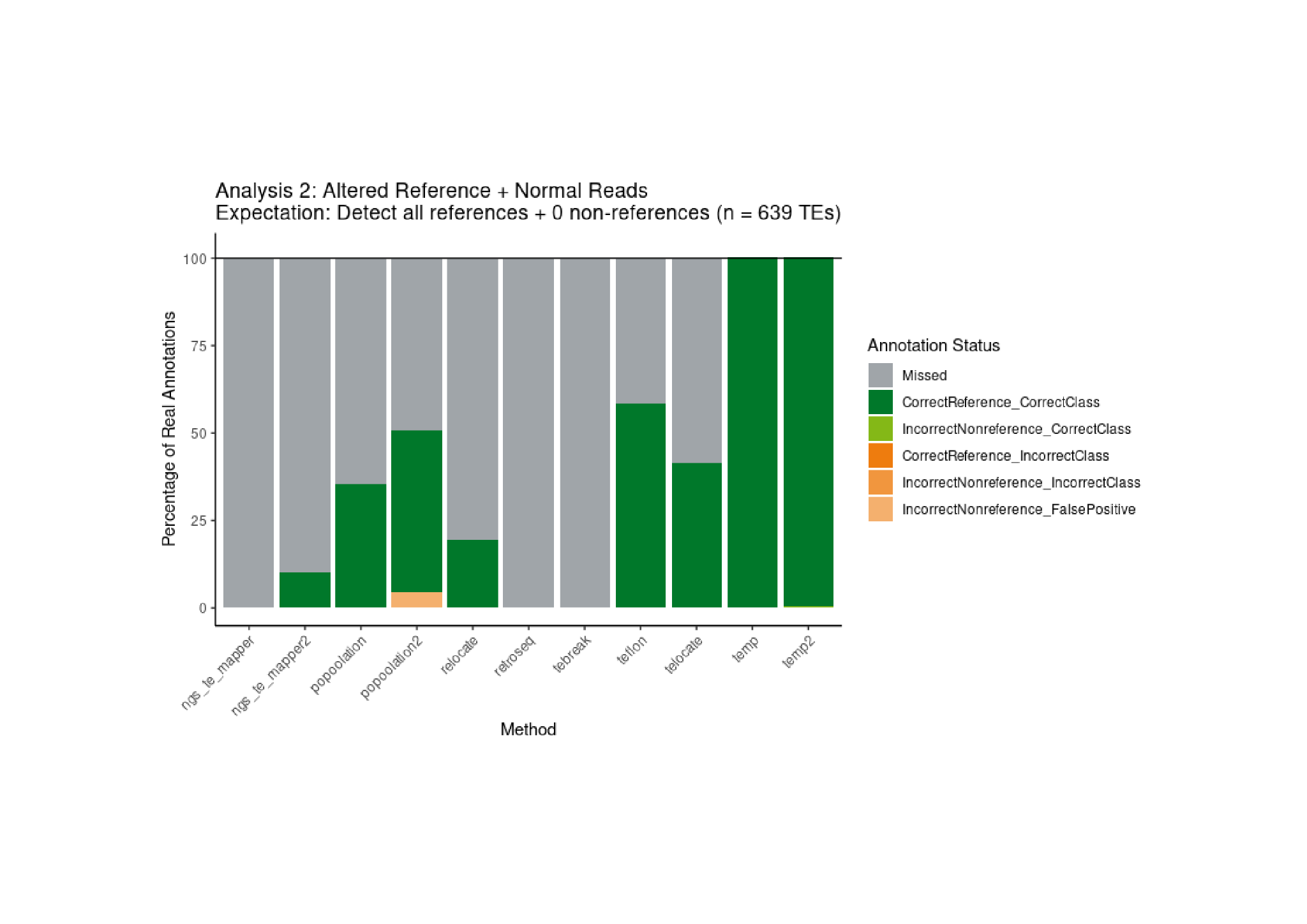


Figure 2. TE benchmarking results for analysis 2. Methodologies were able to detect >100% of the read annotations if loci were annotated multiple times, or if false positives were identified. The status of each annotated locus is indicated by the key.

### **Analyses 3 and 4: Mapping short-read and long-read sequencing datasets from the same isolate to the IPO323 reference genome assembly**

In addition to simulations, it can also be beneficial to benchmark TE detection methods in a real-world context. We therefore aimed to benchmark TE detection methods by comparing TE calls using a short-read dataset to SVs called using a long-read dataset for the same isolate. TEs were detected using the short-read dataset for the isolate CRI10 using McClintock2, whilst SVs were called using the long-read dataset for CRI10 using PBSV (<https://github.com/PacificBiosciences/pbsv>).

Consistent with analyses 1 and 2, the number of TE insertion calls supported by the SV calls was highly variable, ranging from 0 (ngs_te_mapper) to 1,487 (TEFLoN). There are significant differences in the number of supported, dubious, and unsupported TE calls among methodologies (Pearson’s Chi-squared test: 𝝌^2^_22_ = 7431.5, p < 0.01), with TEFLoN and PoPoolationTE showing the highest association with supported calls. For reference TE insertions, two methodologies had over 80% of their TE annotations supported by SVs: PoPoolationTE (86.06%, 809/940 total reference hits) and TEFLoN (83.40%, 1,477/1771 total reference hits). For non-reference TE insertions, levels of support were generally lower, with only two methods having over 50% of their TE annotations supported by SVs: PoPoolationTE (77.54%, 183/236 total non-reference hits) and TEMP2 (59.68%, 111/186 total non-reference hits) (Figure 3; Supplementary Data 14).


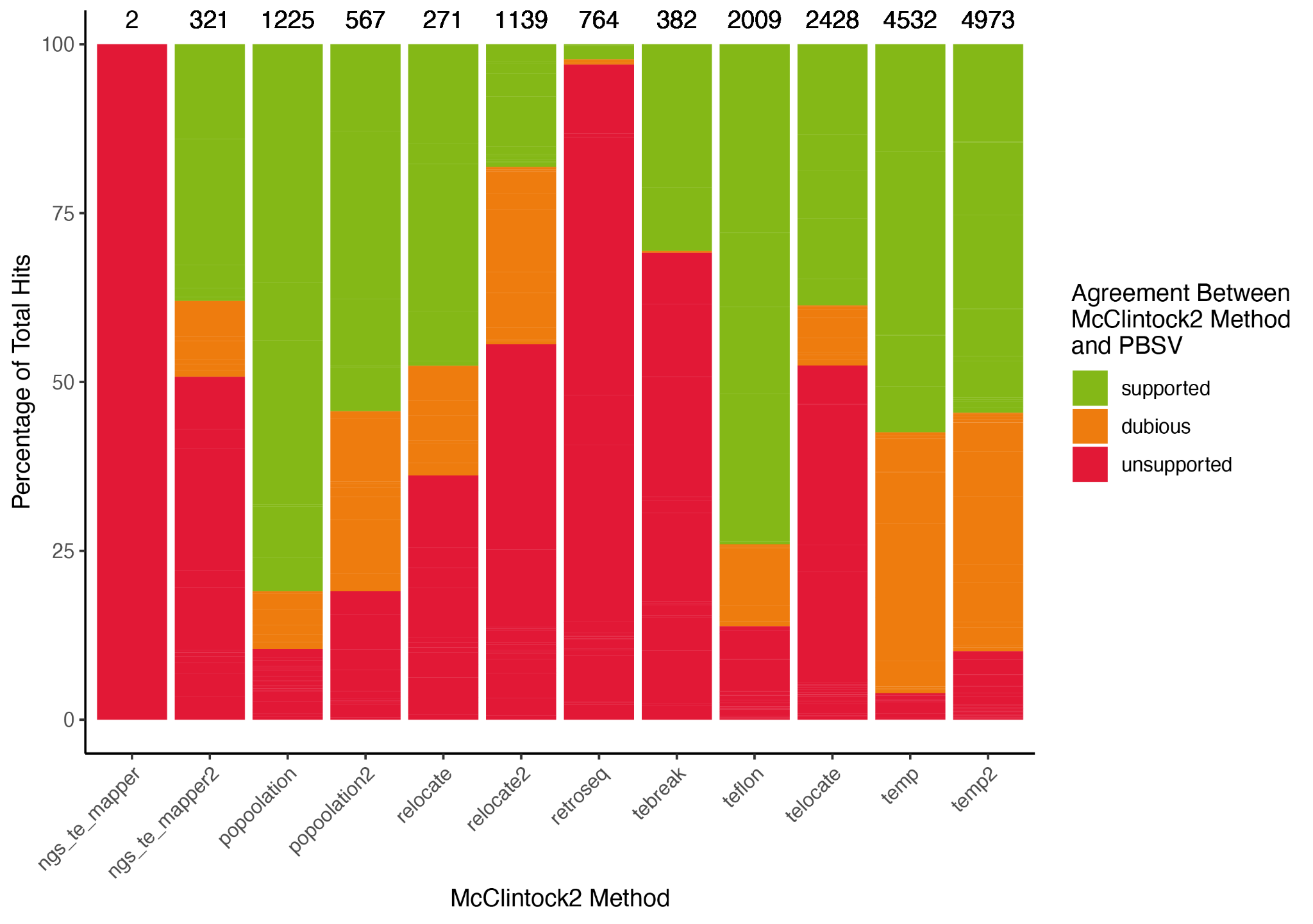


Figure 3. TE benchmarking results for analyses 3 and 4. Numbers at the top of each bar indicate the total number of loci annotated by each methodology. Agreement between each methodology and long-read SV calls are indicated in the key.

We reperformed the above benchmarking analyses with a combined methodology of TEFLoN and PoPoolationTE for all TEs, and TEMP2 for non-reference TEs, and observed an increase in TE detection performance. In analysis 1, 74.37% of TEs were detected (504 TEs: 477 reference TEs; 22 non-reference TEs; 2 reference TEs misclassified as non-reference TEs of the wrong family; 1 reference TE annotated as the correct TE classification, but wrong family; 2 non-reference TEs annotated as the correct TE classification, but wrong family). In analysis 2, 68.34% of TEs were detected (436 TEs: 435 reference TEs; 1 reference TE incorrectly annotated as non-reference, but of the correct TE classification and family). In analyses 3 and 4, 2,302 TEs were detected, of which 80.79% had some level of support from long-read SV calls (1,608 TEs supported; 251 TEs marked as having dubious support), whilst 19.21% were unsupported (442 TEs).

**The impact of sequencing depth on TE detection ability**

The collection of isolates used in this study belong to different collections that have been sequenced with varying iterations of different short-read technologies, in part due to the timing of collection and sequencing. We wanted to assess the consistency of McClintock2 methodologies at varying sequencing depths and with varying short-read technology to determine the impact this variation has on our ability to detect TE insertions. We selected a single dataset from each collection with the highest read count and detected TEs with McClintock2 using the selected combined methodology (see above). Following this, we downsampled each dataset to standardised read counts with the following estimates using seqtk sample (<https://github.com/lh3/seqtk>):

- 9,860,000 reads ~ 31x coverage
- 8,410,000 reads ~ 28x coverage
- 7,480,000 reads ~ 25x coverage
- 6,540,000 reads ~ 22x coverage
- 5,610,000 reads ~ 19x coverage
- 4,670,000 reads ~ 16x coverage
- 3,740,000 reads ~ 13x coverage
- 2,800,000 reads ~ 10x coverage
- 1,870,000 reads ~ 7x coverage
- 935,000 reads ~ 4x coverage

The minimum number of reads required for high-quality TE detection was calculated by determining the minimum number of reads at which the increase in TE locus detection starts to plateau (i.e. the plateau point). The plateau point was calculated by identifying the point at which the change in TE locus count between two points divided by the maximum change in TE locus count was less than 0.1 (Figure 3). Isolates were then labelled as accepted if their read count was above this threshold, borderline if their read count was within 10% of the threshold, and rejected if it was below 90% of the threshold. TE locus annotations at different read depths are provided in supplementary data 14.


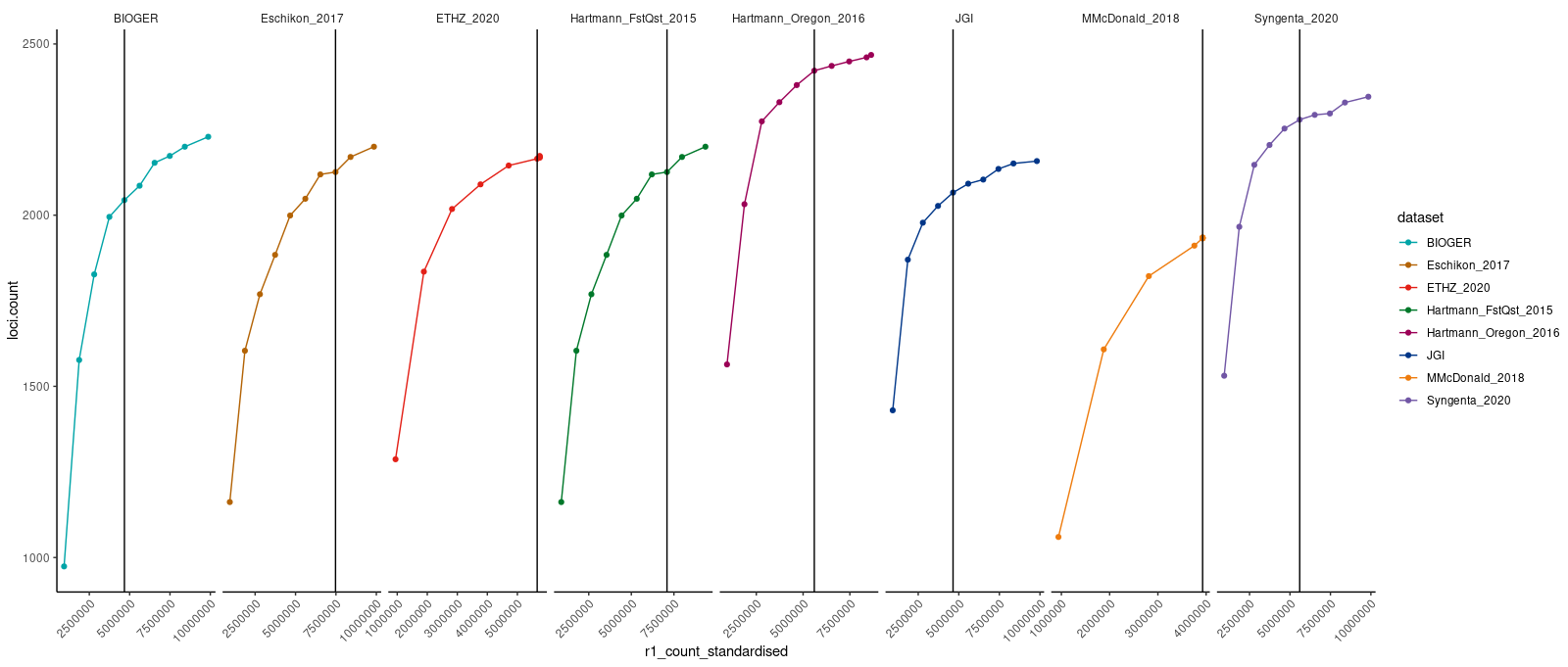


Figure 3. TE locus count for the test isolate from each collection using the whole dataset and downsampled reads. Vertical lines show the mathematically defined plateau point, below which the ability to detect the whole TE population of an isolate begins to diminish.

**Brief Discussion**

Whilst long-read sequencing costs continue to decrease, short-read sequencing continues to be the dominant technology for large-scale population-level studies, as sequencing thousands of individuals continues to be prohibitively expensive. We found significant heterogeneity in the performance of TE annotation tools using short-read data and show that complementary methodologies are required to gain a more complete picture of the total TE repertoire. Given the high variability in TE content among species, it is unlikely that tools will perform consistently across study systems, and we recommend that researchers strongly consider performing benchmarking of methodologies in their own systems before choosing one, or several, methods for their own analyses. The benchmarking analyses we perform here can provide researchers with a framework to determine the best choice of tools for their own research. As sequencing costs continue to fall, long-read sequencing should take precedence in TE research due to their repetitive nature and issues with correctly assembling TE-rich regions from reads that do not span whole elements with flanking host sequence.
