# Supplementary material for: Historic transposon mobilisation waves create distinct pools of adaptive variants in a major crop pathogen
Source: Nat Commun. 2025 Nov 12;16:9961. doi: 10.1038/s41467-025-64944-4 (PMC12612061; doi:10.1038/s41467-025-64944-4)
Supplement: Supplementary file 6 — Reporting Summary [file 41467_2025_64944_MOESM6_ESM.pdf]

nature portfolio

Corresponding author(s): Tobias Baril, Daniel Croll

Last updated by author(s): 2025/07/18

Reporting Summary

Nature Portfolio wishes to improve the reproducibility of the work that we publish. This form provides structure for consistency and transparency in reporting. For further information on Nature Portfolio policies, see our Editorial Policies and the Editorial Policy Checklist.

Statistics

For all statistical analyses, confirm that the following items are present in the figure legend, table legend, main text, or Methods section.

n/a	Confirmed	
☐	☒	The exact sample size (*n*) for each experimental group/condition, given as a discrete number and unit of measurement
☒	☐	A statement on whether measurements were taken from distinct samples or whether the same sample was measured repeatedly
☐	☒	The statistical test(s) used AND whether they are one- or two-sided<br>*Only common tests should be described solely by name; describe more complex techniques in the Methods section.*
☐	☒	A description of all covariates tested
☐	☒	A description of any assumptions or corrections, such as tests of normality and adjustment for multiple comparisons
☐	☒	A full description of the statistical parameters including central tendency (e.g. means) or other basic estimates (e.g. regression coefficient) AND variation (e.g. standard deviation) or associated estimates of uncertainty (e.g. confidence intervals)
☐	☒	For null hypothesis testing, the test statistic (e.g. *F*, *t*, *r*) with confidence intervals, effect sizes, degrees of freedom and *P* value noted<br>*Give P values as exact values whenever suitable.*
☒	☐	For Bayesian analysis, information on the choice of priors and Markov chain Monte Carlo settings
☒	☐	For hierarchical and complex designs, identification of the appropriate level for tests and full reporting of outcomes
☒	☐	Estimates of effect sizes (e.g. Cohen's *d*, Pearson's *r*), indicating how they were calculated

Our web collection on statistics for biologists contains articles on many of the points above.

Software and code

Policy information about availability of computer code

Data collection	No software was used for data collection
Data analysis	We used the R version v4.3.3 with the following packages: ggplot2, LEA, tidyverse , data.table , magrittr, KaryoploteR, qvalue<br><br>Public software used included Earl Grey v3.0, CD-Hit-Est v4.8.1, MAFFT v7.505, T-COFFEE v13.45.0.4846264, EMBOSS v6.6.0, HMMER v3.3.2, REVEAL REcursive Exact-matching ALigner, Trimmomatic v0.40, bowtie2 v2.5.2, GATK4 v4.4.0.0, vcftools v0.1.16, McClintock2 meta-pipeline including: ngs_te_mapper, ngs_te_mapper2, PoPoolationTE, PoPoolationTE2, RelocaTE, RelocaTE2, RetroSeq, TEBreak, TEFLoN, TE-locate, TEMP, and TEMP2; RepeatMasker v4.1.4, ART MountRainier-2016-06-05, pbsv v2.9.0, BedTools v2.31.0, seqtk v1.4-r122, BayPass v2.4, InterProScan v5.67-99.0, vcf2gwas, circos v0.69-8<br><br>Custom code developed for this study is hosted on GitHub: https://github.com/TobyBaril/Baril2025_ZT_TE_PopGen and Zenodo under DOI: 10.5281/zenodo.17021596 (https://doi.org/10.5281/zenodo.17021596)

For manuscripts utilizing custom algorithms or software that are central to the research but not yet described in published literature, software must be made available to editors and reviewers. We strongly encourage code deposition in a community repository (e.g. GitHub). See the Nature Portfolio guidelines for submitting code & software for further information.

Data

Policy information about availability of data

All manuscripts must include a data availability statement. This statement should provide the following information, where applicable:

- Accession codes, unique identifiers, or web links for publicly available datasets
- A description of any restrictions on data availability
- For clinical datasets or third party data, please ensure that the statement adheres to our policy

The data underlying this article are available in the article and in its online supplementary material on Zenodo (DOI: 10.5281/zenodo.15119623). The manually-curated TE library is available from Zenodo (DOI: 10.5281/zenodo.8379980), and has been submitted to the Dfam consortium (Dfam Release 3.9).

Research involving human participants, their data, or biological material

Policy information about studies with human participants or human data. See also policy information about sex, gender (identity/presentation), and sexual orientation and race, ethnicity and racism.

Reporting on sex and gender	not applicable
Reporting on race, ethnicity, or other socially relevant groupings	not applicable
Population characteristics	not applicable
Recruitment	not applicable
Ethics oversight	not applicable

Note that full information on the approval of the study protocol must also be provided in the manuscript.

Field-specific reporting

Please select the one below that is the best fit for your research. If you are not sure, read the appropriate sections before making your selection.

☐ Life sciences ☐ Behavioural & social sciences ☒ Ecological, evolutionary & environmental sciences

For a reference copy of the document with all sections, see nature.com/documents/nr-reporting-summary-flat.pdf

Ecological, evolutionary & environmental sciences study design

All studies must disclose on these points even when the disclosure is negative.

Study description	Genomic characterization of transposable element variation in a global set of fungal pathogen strains from infected wheat fields.
Research sample	We analyzed a global collection of genomes of the fungal wheat pathogen Zymoseptoria tritici from field-collected isolates established with the aim to cover most of the currently known area where this pathogen is found. The collection contains isolates from all the Americas, North Africa, the Middle-East, Oceania and Europe. Isolate collection years span over the last 30 years (earliest recorded sampling in 1989). All resequencing is available online from the NCBI SRA database.
Sampling strategy	Most samples were collected in a hierarchical manner with multiple isolations made for individual wheat fields. Different samples per wheat field were defined as originating from different wheat leaves to reduce the likelihood of including clonal genotypes. We aimed to cover the global geographical range of this pathogen and sequenced as many genomes as isolates could be made available, while limiting the sequencing of a single field to typically below 30. We used all publicly available genomic sequence data of the pathogen where appropriate.
Data collection	Information on the date/year and site of collection was recorded in the field by co-authors of the original publications reporting the genome datasets. No specific instruments were used for this recoding (aside from paper, pen, and computers).
Timing and spatial scale	Sampling was performed on a world-wide scale. Sampling during wheat-growing seasons spanned multiple decades (1980-2010s), depending on the available collections from all co-authors of the original publications. Detailed location and sampling years are available as a supplementary material, however the month of collection was not always recorded. Individual fields were only sampled once per growing season and only a small set of samples originate from the same field collected in different years. Supplementary Tables identify sampling year and location.
Data exclusions	Genomic datasets failing minimum quality criteria of copy-number variation calling were excluded regardless of their provenance.
Reproducibility	Not applicable as no experiments were conducted. Association mapping studies were performed using environmental data at the time of collection using all retained samples.
Randomization	No randomization was performed as no experiments were conducted. Genetic substructure in the dataset was controlled for using

mixed linear models with a kinship matrix to avoid inflated G-E associations.

Blinding	No blinding was performed as no experiments were conducted to assess trait values. Association mapping was performed using environmental data from WorldClim

Did the study involve field work? ☐ Yes ☒ No

Reporting for specific materials, systems and methods

We require information from authors about some types of materials, experimental systems and methods used in many studies. Here, indicate whether each material, system or method listed is relevant to your study. If you are not sure if a list item applies to your research, read the appropriate section before selecting a response.

Materials & experimental systems

n/a	Involved in the study
☒	☐ Antibodies
☒	☐ Eukaryotic cell lines
☒	☐ Palaeontology and archaeology
☒	☐ Animals and other organisms
☒	☐ Clinical data
☒	☐ Dual use research of concern
☒	☐ Plants

Methods

n/a	Involved in the study
☒	☐ ChIP-seq
☒	☐ Flow cytometry
☒	☐ MRI-based neuroimaging

Plants

Seed stocks	n/a
Novel plant genotypes	n/a
Authentication	n/a
